# Study on Holocene environmental evolution based on grain size end-member model: A case study of two outcrop sections in Salawusu River Basin

**DOI:** 10.1371/journal.pone.0305282

**Published:** 2024-09-20

**Authors:** Dongfeng Niu, Tong Li, Yuanyu Zhong, Longlong Liu, Baosheng Li

**Affiliations:** 1 Department of Geography, Lingnan Normal University, Zhanjiang, China; 2 Department of Geography and Spatial Information Techniques, Ningbo University, Ningbo, China; 3 School of Geography, South China Normal University, Guangzhou, China; University of California Santa Cruz, UNITED STATES

## Abstract

Samples from two outcrop sections, MGS1 and DGS1 of Milanggouwan and Dishaogouwan in the Salawusu River Basin, were studied in terms of grain size using end-member model. Results show that: 1) MGS1 layer particles are more concentrated, better sorting, and smaller skewness and kurtosis values than those of DGS1. Whereas in the upper part of the DGS1 section, the grain size of the paleodune is coarser, with better sorting and sharper peak, comparing with the lower lacustrine sediments. 2) Three end-member components, EM1 (end-member 1), EM2 (end-member 2) and EM3 (end-member 3), which reflect sedimentary dynamic characteristics, are extracted by end-member analysis. The EM1 indicates the hydrodynamic force with great variation, EM2 indicates transporting force by flowing water and EM3 indicates the depositional environment closely related to the wind activity. 3) According to the accumulation processes of MGS1 and DGS1 strata, a total of four climate periods can be identified, namely early warming period, Holocene peak period, fluctuating transition to cold period and unstable cooling period. Moreover, EM1 of MGS1 and DGS1 is basically consistent with both the sea surface temperature (SST) in the western tropical Pacific and global temperature trends during the Holocene, suggesting that the environmental fluctuations recorded by MGS1 and DGS1 can be correlated with each other.

## 1 Introduction

The Holocene, the interglacial period in which we currently live, is the latest episode in geological history [1]. Generally, the end time of the Younger Dryas event (about 11.7kaBP) is regarded as the beginning of the Holocene [2]. Although it has lasted only a short time since then, in recent years, many scholars have adopted historical documents [3], loessal-paleosol sequence [4–6], lake deposits [7–9], ice core [10–12], peat [13–15], stalagmite [16–18], tree ring [19–21], and other carriers, found that the Holocene environment has obvious cold, warm, dry and wet fluctuations. The study of Holocene climate change not only provides the long-term time window of past climate change for assessing modern global warming under the background of natural modern interglacial period [10, 12], but also provides important scientific bases for human beings to formulate coping strategies with climate change [13, 15].

The Salawusu River basin is located in the ecologically fragile zone of the boundary between desert and loess in northern China, and its environment is very sensitive to global changes, making it an ideal area for studying environmental evolution. Previous authors have used major elements [22–24], trace elements [25–27], CaCO_3_ [28–30], pollen [31], grain size characteristics [30, 32] and vertebrate fossils [33, 34] to preliminarily reveal the Holocene climatic cold and warm changes recorded in MGS1 and DGS1 section respectively. Due to the great differences in the composition and structure of sedimentary facies between these two sites, MGS1 is interbedded with limnetic facies or fluvial facies and ancient mobile dune sand, whereas DGS1 is dominated by dune sand facies in the upper part and limnetic facies in the lower part of the Dagouwan Formation. There are still many questions about how to relate the environmental fluctuations recorded between them, which is the target discussed in this paper.

The end-member model, proposed by Weltje [35] and improved by Paterson and Helslop [36] plays an important role in reflecting the fractal structure characteristics of complex sediments and revealing provenance and sedimentary dynamic information [37–40]. In this paper, the Holocene strata, MGS1 and DGS1 layers of Milanggouwan and Dishaogouwan, were respectively selected as research objects, and the grain size parameters of them were compared and analyzed. The end-member model was used to decompose the dynamic components affecting the sedimentary differences, so as to explore the Holocene climatic fluctuations recorded between different sedimentary facies of MGS1 and DGS1. This paper was designed to provide a basis for understanding the Holocene environmental evolution in this region.

## 2 Background

**Fig 1:**
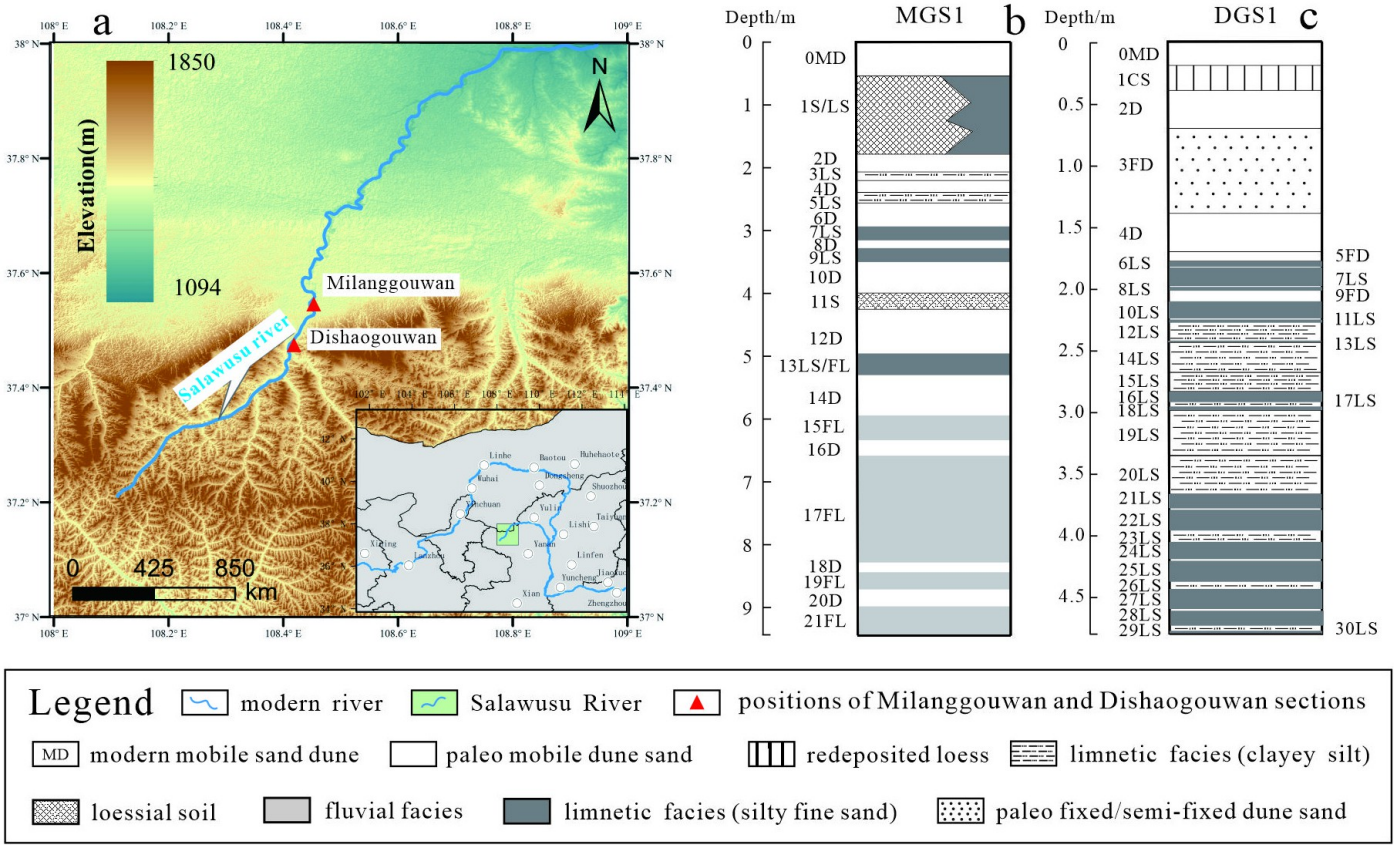
Geographical location and study area of Salawusu River. (a) Location map of the Salawusu River Valley; (b) Vertical facies profiles of MGS1; (c) Vertical facies profiles of DGS1.

The Salawusu River basin is located in the south of the Mu Us Desert in the Ordos Plateau of Inner Mongolia, with Loess Plateau to the southeast and Mu Us desert to the northwest (Fig 1a and 1b). The geographical coordinates are 37°10’ N~37°58’ N, 108°10’ E~108°58’ E (Fig 1b). In terms of climate, it belongs to the semi-arid monsoon climate zone in eastern Inner Mongolia between the Gan-Xin temperate arid zone and the semi-humid temperate zone in North China [41]. The average annual precipitation in this region is 350~400 mm. In the winter half of the year, the wind prevails in the northwest, whereas in summer the wind prevails in the southeast. From old to new, the Holocene Milanggouwan strata can be divided into Dagouwan Formation (Q^1_2^_4_) in the middle and lower part, and Dingshaogouwan Formation (Q^3^_4_) in the upper part [42].

The Milanggouwan profile where involves MGS1 is located on the left bank of the Salawusu River, about 500 m northeast of Milanggouwan Village in the middle reaches of the Salawusu River, with geographical coordinates of 37°45’ 47.2" N and 108°33’ 05.4" E (Fig 1c). The elevation of the top section is about 1313 m, and the accumulation depth of the MGS1 layer is 0~9.44 m (Fig 1d). It consists of 1 layer of modern mobile dune sand (MD), 10 layers of paleo-mobile dune sand (D), 4 layers of fluvial facies (FL), 5 layers of limnetic facies (LS) and 2 layers of paleosoil facies (S) (Fig 1d). Lacustrine facies or fluvial facies overlapped with the interbedded paleo-mobile dune sand, constituting 11 sedimentary cycles. The Dishaogouwan profile where involves DGS1 is located on the left bank of the river in Dishaogouwan Village in Salawusu River Basin, with the geographical coordinates of 37°43’ 26.3" N and 108°31’ 2.3" E (Fig 1c). The elevation of the top section is about 1309 m, and the accumulation depth of DGS1 layer ranges from 0 to 4.84 m (Fig 1e). It includes 1 layer of modern mobile dune sand (MD), 2 layers of paleo-dune sand (D), 3 layers of paleo fixed/semi-fixed dune sand (FD), 12 layers of limnetic facies (LS) and 1 layer of sandy loess facies (CS) (Fig 1e).

## 3 Materials and methods

### 3.1 Sample collection and testing

The sampling interval was determined according to the thickness of the sedimentary layer in the section. A total of 151 samples were collected from the MGS1 layer with a spacing of 3~7 cm and a total of 250 samples were collected at intervals of 1~2 cm in the DGS1 layer. Particle size analysis was completed in the Sediment Laboratory of the School of Geographic Sciences, South China Normal University, using the Mastercizer2000M laser particle size analyzer produced by Malvern Company in the UK, with a analysis range of 0.02~2000 μm. The particle size was expressed as its logarithmic Φ value, and the conversion formula was the Rum-Blen formula. The Folk-Ward graphical method [43] was used to calculate the mean particle size (Mz), standard deviation (σ1), skewness (Sk_1_) and kurtosis (K_G_).

**Fig 2:**
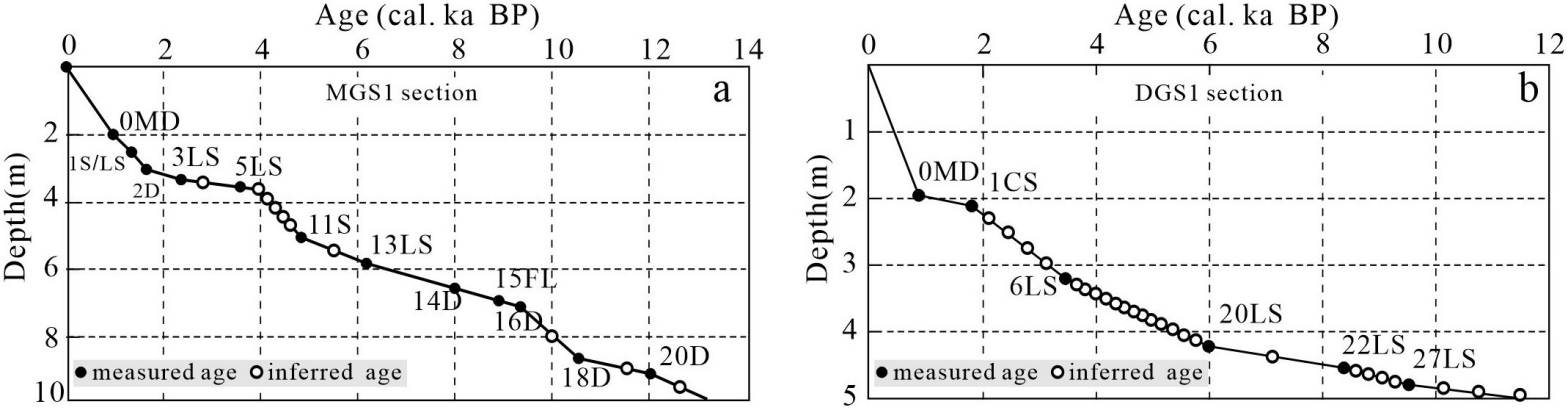
Ages of MGS1 (a) and DGS1 (b) sections.

A total of 12 MGS1 samples were dated (Fig 2a). 8 of them are ^14^C, 2 thermoluminescence (TL) ages and 2 optical stimulated luminescence (OSL) ages. A total of 6 samples were dated in DGS1 (Fig 2b), 5 of them are ^14^C and 1 TL age. The ^14^C dates were determined by the ^14^C laboratory of Cold and Arid Regions Environmental and Engineering Research Institute, Chinese Academy of Sciences.TL dates were determined by thermoluminescence Laboratory, Guangzhou Institute of Geochemistry, Chinese Academy of Sciences. The test material for OSL age was quartz particles <10μm, which were determined by the Opto-luminescence Laboratory of the Institute of Hydrogeology and Engineering Geology, Ministry of Geology and Mineral Resources, China.

### 3.2 End-member method

The theory of end-member modeling is based on the fact that sediments are formed by different material sources or different transport forces through the mixing of components [37–40]. The least square method is used to measure the quality of the model for the sediment grain size data, and then the non-negative matrix is used to decompose, and the distribution and abundance of each endmember are estimated through the iterative process [44]. Each endmember component under different dynamic actions can be separated [36]. The non-parametric method is to separate several particle size components with the same frequency distribution by fitting the whole samples. Parameter method is to fit a single sample, set the best parameters, and then separate each subcomponent of the sample [45]. In this paper, AnalySize-masters software package [36] was run in MatlabR2019a to conduct end-member analysis on imported granularity data. Non-parametric and parametric methods are used respectively (the parametric method adopts the Gen. Weibull distribution function) to conduct end-member analysis on MGS1 and DGS1 granularity data under the assumption of the number of end-members ranging from 1 to 10, and then compare the linear correlation, angle deviation, end-member correlation and other parameters to select an appropriate method for end-member decomposition [46, 47].

## 4 Results

### 4.1 Grain size content

**Fig 3:**
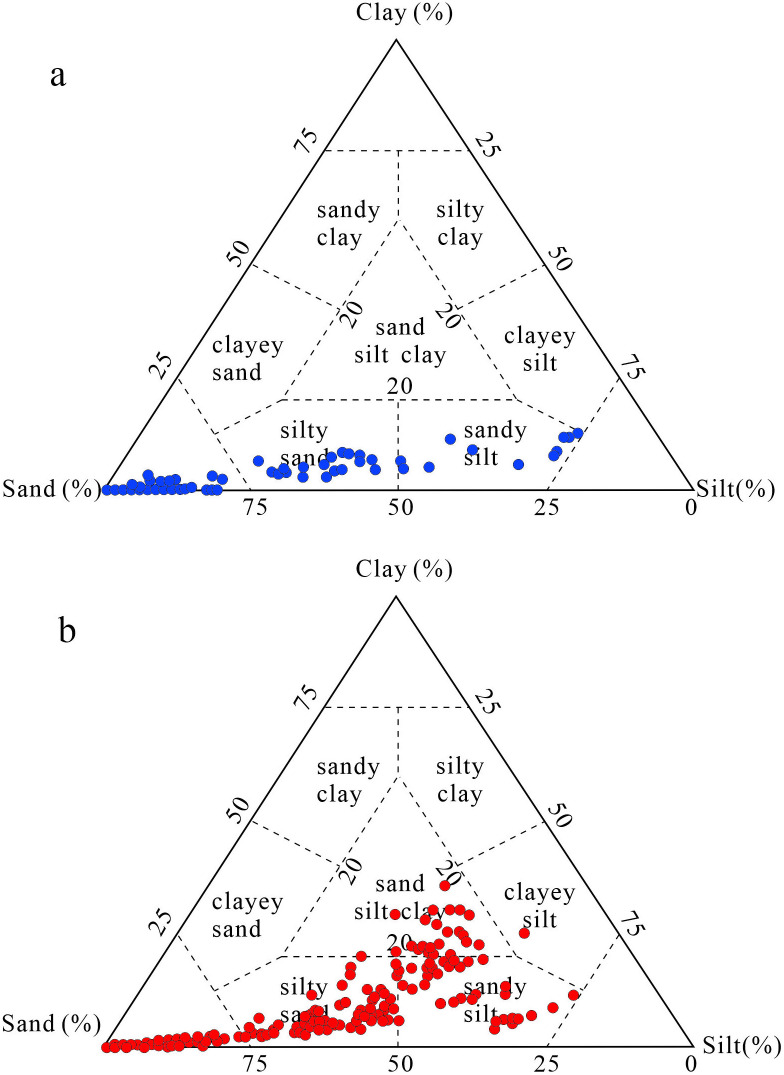
The ternary diagram of MGS1 (a) and DGS1 (b) according to Sheppard [48].

Sheppard sediment classification divides sediment types according to the proportion of sand, silt and clay composition [48]. By classifying MGS1 and DGS1 samples from each layer, it can be found that MGS1 sediments are mainly distributed in the range of sand, silty sand and sandy silt (Fig 3a). In addition to the distribution of DGS1 sediments in the above range, some samples are also distributed in the mixed range of sand silt clay in the middle (Fig 3b), indicating that the particle composition of DGS1 is more complex than that of MGS1, and the same layer may be mixed with substances of various dynamic processes.

**Fig 4:**
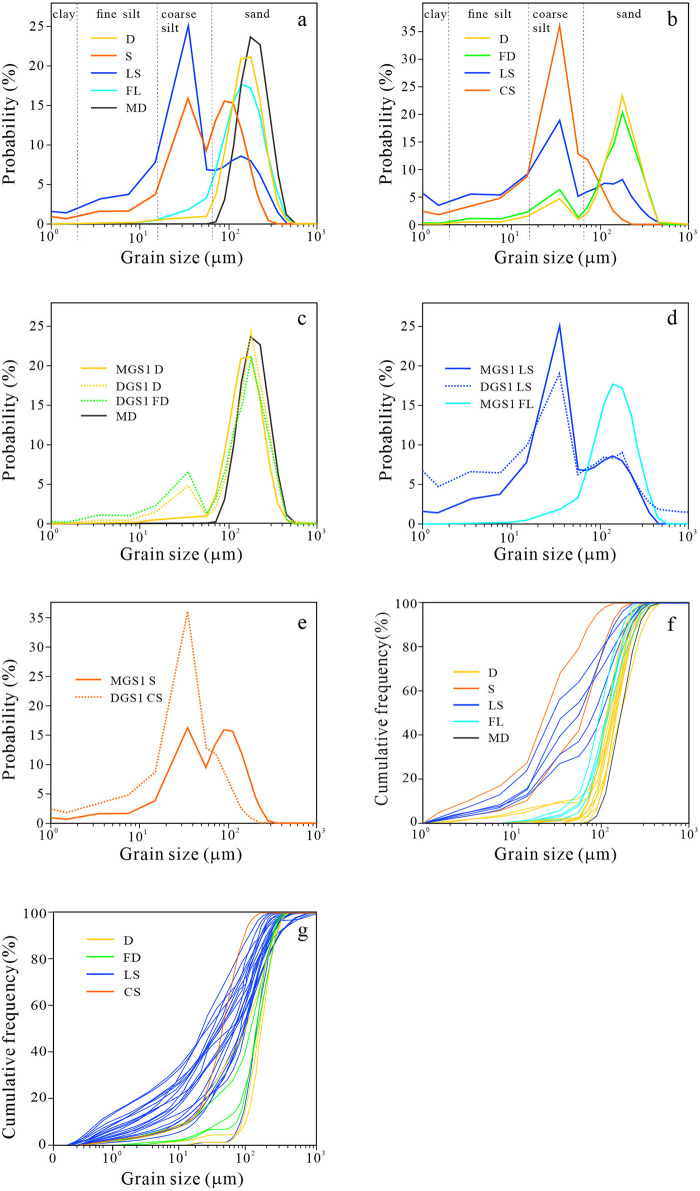
Grain size frequency distribution curve and cumulative frequency curve of each layer of MGS1 and DGS1. Grain size frequency distribution of the whole layers of MGS1 (a) and DGS1 (b). (c) Comparison of grain size frequency distribution of dune sand facies (D, FD and MD). (d) Grain size frequency distribution in LS and FL facies. (e) Frequency distribution of S and CS grain size. Cumulative frequency curve of MGS1 (f) and DGS1 (g).

From the grain size frequency distribution of each segment of the two sections, the D, MD and FL facies of MGS1 all show a single peak shape, with the peak value in the sand range, and the material composition is relatively simple. Whereas the LS and S facies show double peaks, with the main peak located in the coarse silt range and the secondary peak located in the sand and gravel range. The material composition is relatively complex (Fig 4a). In DGS1, the D, FD, and LS facies all show double peaks, with the main peak of dune sand in the gravel range and the secondary peak in the coarse silt range. The main peak of lacustrine facies is coarse silt, and the secondary peak is in the range of sand and gravel (Fig 4b).

From the comparison of particle size frequency distribution (Fig 4c), it was found that the dune sand particle sizes of MGS1 and DGS1 were concentrated in the range of 170~180 μm. The difference is that MGS1 has an obvious single peak, while DGS1 has a secondary peak at 35 μm for D and FD sand (Fig 4c). The agglomeration and sorting degree of sand facies of MGS1 and DGS1 are not as good as that of modern mobile sand.

The LS of MGS1 and DGS1 is the main peak at 32 μm and the secondary peak at 139 μm (Fig 4d). The aggregation degree of MGS1 at the main peak is better than that of DGS1, and DGS1 contains more fine particles such as clay and fine silt. According to Yin Zhiqiang ’s study on the distribution characteristics of grain size components in lakes in northern China [49], the frequency distribution curves of MGS1 and DGS1 facies correspond to the lakeshore—central transition phase, but the particles are coarser than those of ordinary lake deposits, indicating large lake disturbance or large particle size of provenance. Among them, the variation of hydrodynamics indicated by the LS of DGS1 was larger. The mode particle size of MGS1 fluvial phase is 142 μm, with obvious unimodal peak, and the particle size of the dominant component is 100~200 μm, which corresponds to the floodplain sedimentary environment [49].The MGS1 and DGS1 S facies (Fig 4e) have relatively fine grain size. The S facies of MGS1 is black loessial soil, with double peaks, and the peak values are 36 μm and 90 μm. The secondary loess facies of DGS1 showed a single peak, with the peak value located at 35 μm, and highly clustered in the coarse silt range.

The cumulative frequency curves of MGS1 and DGS1 (Fig 4f and 4g) showed that the sorting property of the two profiles gradually deteriorated from MD, FL, S to LS facies. The LS frequency curve of MGS1 is steep, while that of DGS1 is gentle, indicating that the material composition is more complex. At the same time, the lacustrine phase of DGS1 accumulates more fine particles, and the suspended load component is significantly higher than that of MGS1.

### 4.2 Comparison of particle size parameters

**Fig 5:**
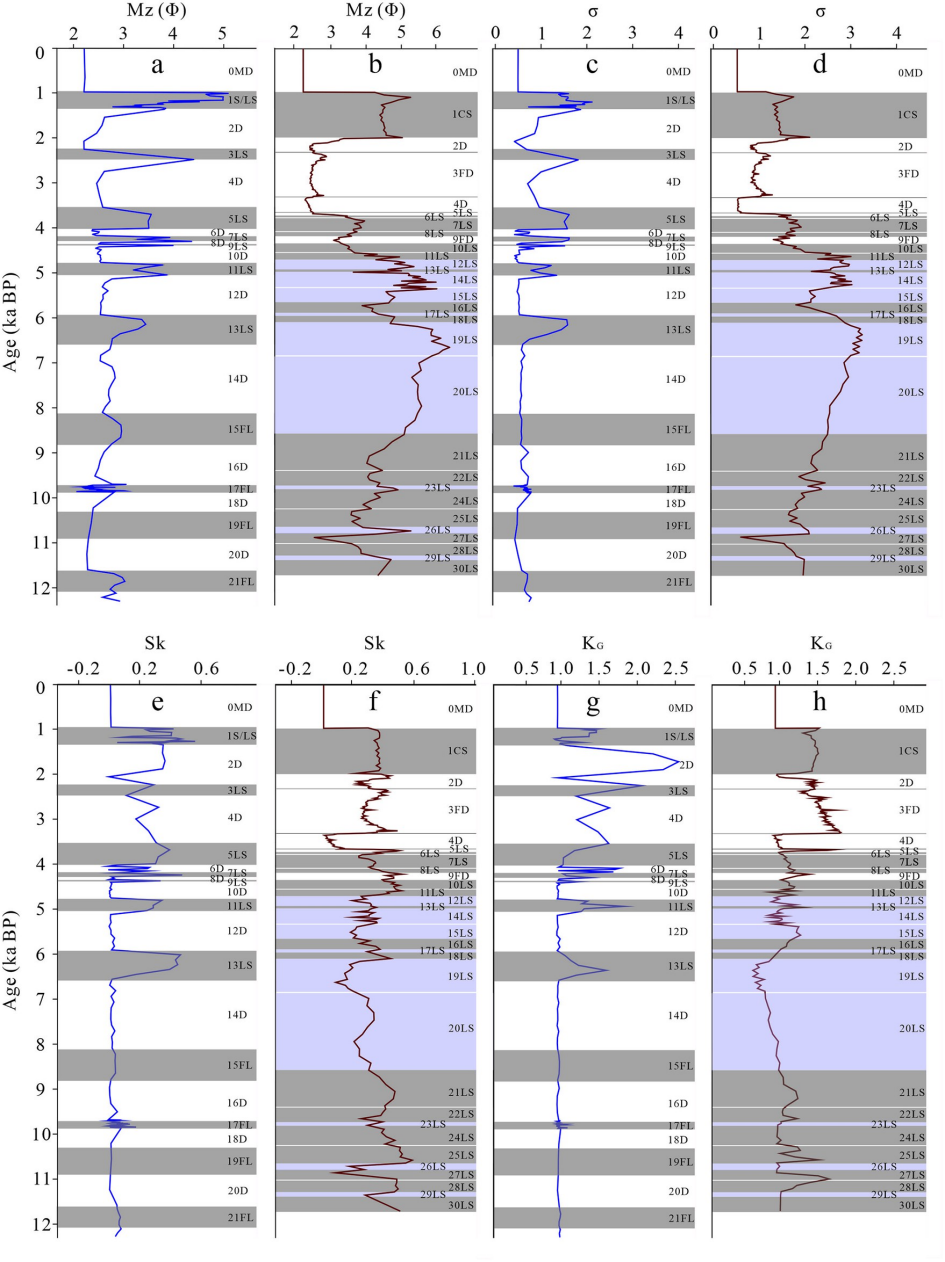
Comparison of facies and particle size parameters between MGS1 and DGS1 layers. Detailed information of a~h please refer to the text.

The grain size parameter of sediment can reflect the sedimentary environment and moving force, and it is one of the important indicators to reflect the climate change [45]. Mean particle size (Mz) represents the thickness of sediment particles, which is an index reflecting the strength of moving and operating force [32]. The Mz of the whole layer of MGS1 is 2.91 Φ, ranging from 2.05~5.09 Φ (Fig 5a), and that of DGS1 is 3.69 Φ, ranging from 2.27 Φ to 6.38 Φ (Fig 5b). It can be seen that DGS1 is finer than that of MGS1 in general, but the variation range of different layers is larger than that of MGS1.The Mz of the sand phase of the two sections is similar, and the particle size difference is less (Fig 5a and 5b).

The standard deviation (σ) indicates the degree of sediment sorting [28]. σ of MGS1 profile shows that the sorting property gradually deteriorates from the D, FL, LS to S facies (Fig 5c). The sorting of DGS1 from MD, FD, CS to LS facies gradually weaken (Fig 5d). The σ of the D sand of MGS1 is 0.62, and that of DGS1 is 0.82 and 1.04, respectively. It can be seen that the sorting of the D sand of MGS1 is better. The σ of MGS1 is 1.36, and that of DGS1 is 2.28, indicating that MGS1 has better sorting degree.

Skewness (Sk) represents the degree of symmetry of sediment thickness distribution [45]. The Sk of D of MGS1 is nearly symmetric, except for 2D, 4D and 6D, which are positively skewed and extremely positively skewed (Fig 5e). The FL are nearly symmetrical. The LS are mainly positive and polar positive. The 0MD and 4FD of dune sands in DGS1 are nearly symmetric, and the FD dune sands are positively skewed and extremely skewed. The LS and CS sand are positively skewed and extremely skewed.

Kurtosis (K_G_) reflects whether the sediment is normally distributed [37]. 2D, 4D, and 6D in MGS1 were characterized by sharp and very sharp kurtosis, whereas the rest have moderate kurtosis. The FL facies is moderate kurtosis; LS and S facies are characterized by sharp kurtosis (Fig 5g). The 0MD and 4FD in DGS1 have moderate kurtosis (Fig 5h). Both FD sand and CS sand have sharp kurtosis. In the limnetic facies phase, the kurtosis of 6LS~18LS and 21LS~30LS is moderate and sharp, whereas that of 19LS and 20LS is flat.

### 4.3 End-member analysis results

Only the comparison of grain size composition and grain size parameters cannot well reveal the internal reasons for the differences in sediment grain size. Only by analyzing the various information of provenance, dynamics and deposition processes mixed in the grain size data, can the grain size data be endowed with richer environmental significance [50]. When the linear correlation is larger, the angle deviation and the correlation degree of end members are smaller, indicating that the fitting degree of end members is better, and the sensitive grain level of the dynamic characteristics of the profile can be extracted [50].The non-parametric and parametric methods were used to analyze the MGS1 and DGS1 data, which showed that the non-parametric method had a better fitting degree and was in line with the particle size characteristics of the overall sample. Therefore, the non-parametric method was selected.

**Fig 6:**
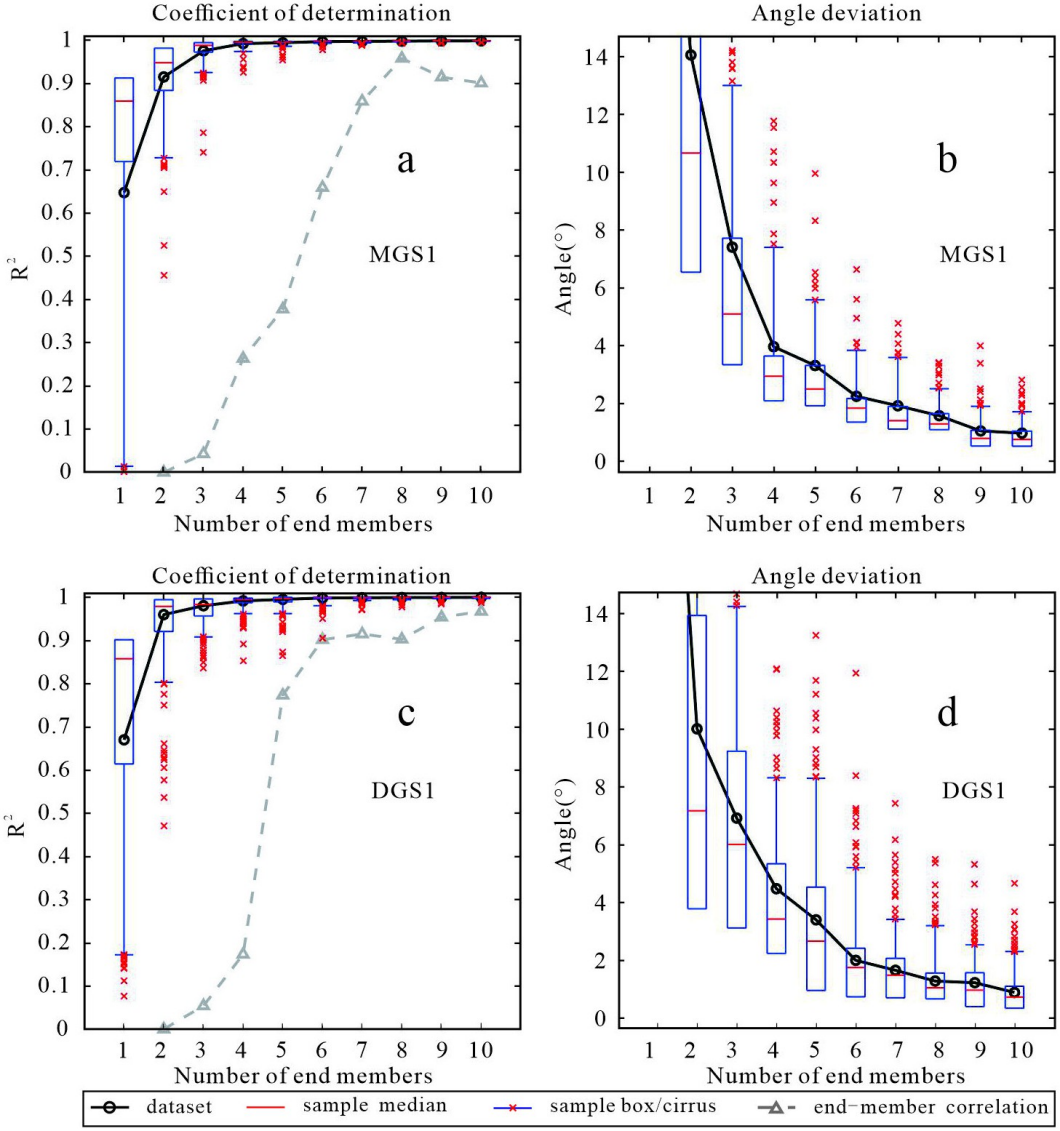
Non-parametric endmember decomposition results and discriminant indexes of MGS1(a, b) and DGS1(c, d).

By comparing the number of each end-member, it was found that as the number of end-members increases, both the linear correlation (Fig 6a and 6c) and the angle deviation (Fig 6b and 6d) fitting results are better (Table 1). Although when the end-member is 3, the correlation between each end-member is relatively poor, it conforms to the principle of selecting the least end-member number when the fitting degree is satisfied. Therefore, in this paper, the profile granularity data is decomposed into three end members.

**Table 1:** Fitting results of different number of endmembers.

section	endmember number	linear correlation	angle deviation (°)	endmember correlation
MGS1	3	0.97	7.4	0.043
	4	0.99	4.0	0.265
	5	0.99	3.3	0.563
DGS1	3	0.98	7.2	0.054
	4	0.99	4.8	0.167
	5	0.99	3.7	0.802

**Fig 7:**
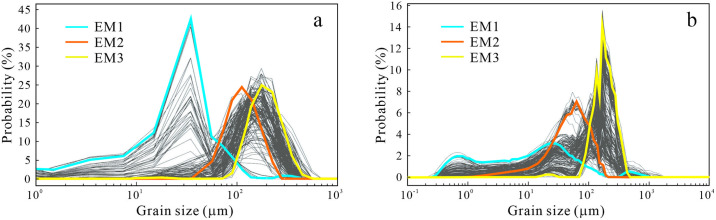
End-member frequency distribution curves of MGS1 (a) and DGS1 (b).

The three curves (in blue, orange and yellow respectively) in the end-member frequency distribution curve (Fig 7) respectively represent the three end-members, namely the dynamic component. The average particle size of the three end-members of MGS1 is 22.83 μm, 101.56 μm and 171.01 μm, respectively (Table 2). As shown in the table, EM1 has poor sorting, negative skewness and sharp kurtosis, with silt accounting for 74.62%, sand accounting for 14.84% and clay accounting for 10.58%, respectively (Table 2), indicating that this end-member has a variety of sources, and EM1 accounts for the largest proportion in limnetic phase (Table 3). EM2 and EM3 are better than EM1, with nearly symmetrical skewness, medium kurtosis and sand accounting for more than 90%. The silt content of EM2 is 9.55%, while that of EM3 is only 0.38%, indicating that the dynamic process of EM2 is more complex than that of EM3.

**Table 2:** Grain size parameters and composition characteristics of each endmember for MGS1 and DGS1.

Profile	Endmember	Grain size parameters				Grain compositions (%)		
		Mz/μm	σ1	Sk_1_	K_G_	Clay	Silt	Sand
MGS1	EM 1	22.83	3.07	-0.27	1.37	10.58	74.62	14.79
	EM 2	101.56	1.41	-0.03	0.93	0.00	9.55	90.45
	EM 3	171.01	1.43	0.04	0.93	0.08	0.38	99.54
DGS1	EM 1	7.57	5.89	-0.21	0.81	34.44	55.46	10.10
	EM 2	43.46	2.32	-0.28	1.16	2.68	61.39	35.93
	EM 3	165.93	1.48	0.01	0.95	0.07	1.28	98.65

**Table 3:** Endmember component contents of each layer of MGS1 and DGS1.

Profile	Facies	EM1(%)	EM2(%)	EM3(%)
MGS1	D	3.10	38.65	58.25
	S	52.10	47.90	0.00
	LS	65.50	14.05	20.45
	FL	8.18	70.63	21.20
	MD	0.00	0.00	100.00
	The whole interval	23.13	36.42	40.45
DGS1	MD	15.27	7.58	77.15
	FD	10.64	16.84	72.52
	LS	40.26	34.46	25.27
	CS	26.04	73.96	0.00
	The whole interval	34.52	31.43	34.05

The average particle sizes of the three end-members of DGS1 were 7.57 μm, 43.46 μm and 165.93 μm, respectively (Table 3), which are all smaller than those of MGS1. EM1 has a poor sorting range, negative skewness, flat kurtosis, mainly silt and clay, and a large amount of fine particulate matter (Table 2), which occupies the largest proportion in the limnetic phase (Table 3), indicating that this endmember component also indicates a hydrodynamic process with a large variation, but it is different from the EM1 of MGS1.EM2 is poorly sorted and negatively skewed, mainly composed of silt and sand, and occupies a large proportion in the secondary loess phase. The degree of EM3 sorting is better than that of the former two, and the skewness is almost symmetric, with sand as the main component and sand accounting for 98.65%, which is relatively large in mobile dune sand phase and semi-fixed dune sand phase.

The major end-member component of MGS1 is EM3, subsequently followed by EM2 and EM1 (Table 3). However, the proportion of 3 end-member components of the whole DGS1 section are quite close (Table 3). It can be concluded that MGS1 is more significantly affected by one of the dynamic factors, but also partially affected by the other two. However, DGS1 was affected by different dynamics to an average degree.

## 5 Discussion

### 5.1 The environmental significance of granular endmember indication

The decomposed end-members can indicate different environmental controlling factors [50]. The average particle size of EM1 in MGS1 is 22.83 μm, and the mode particle size is 35 μm (Table 2), belonging to the suspended component. At the same time, the clay content in MGS1 is the highest among the three end-members, and it is also mixed with a large amount of silt and a certain amount of sand, and the sorting is poor, and it is dominant in the limnetic phase and paleosoil phase. The components with particle size of 10~70 μm in the lake sediments belong to the aeolian sediments formed by the coarse-suspended components of loess and sandstorm dust and then transformed by flowing water, which are also found in the sediment samples of some other lakes in northern China, such as Dali Lake and Bayanchagan Lake [49]. Therefore, it can be inferred that the material source of EM1 mainly is terrigenous debris such as sand dunes, then through the mixture of water and wind into the lake, forming lacustrine sediments. Or in the low-lying areas, fine particulate matter through plant breeding and form dark loessial soil. Therefore, EM1 indicates the improvement of climate conditions, characterized by plentiful rainfall and rivers into the lake. The average particle size of EM2 of MGS1 is 101.56 μm, the mode particle size is 112.5 μm, and it is composed of 9.55% silt and 90.45% sand, accounting for the largest proportion in the fluvial phase (Table 2). In this phase, the peak value is about 100 μm and the sorting is poor, which indicates the sedimentary process of floodplain with weak hydrodynamic conditions [49]. The particle size composition and particle size parameters of EM3 of MGS1 are very similar to those of DGS1. The average particle size of EM3 of MGS1 is 171.01 μm, and the average particle size of DGS1 is 165.93 μm. The sorting degree is better than other end-members, and the skewness is nearly symmetric. Li et al. found that aeolian sand encroachment desertification is dominated by medium-fine sand (particle size 140~310 μm), with low clay content (less than 5%), and has a positively skewed and narrow peak grain size distribution curve [51], which is consistent with the characteristics of EM3 in this study. Therefore, it can be inferred that EM3 of MGS1 and DGS1 indicates the accumulation of aeolian sand formed during wind transport, which corresponds to the arid environment strengthened by the East Asian winter monsoon. According to the study of dune morphology [52, 53], the sorting degree gradually improved from the bottom to the top of the dune, and the EM3 sorting degree of MGS1 was better, and the content of clay and silt was lower, indicating that MGS1 was located in the relatively convex or top of the dune to receive deposition, whereas DGS1 was in a relatively low-lying sedimentary environment.

The average particle size of EM1 in DGS1 was 7.57 μm, and the three peaks were 0.167 μm, 26.7 μm and 423.8 μm, respectively (Fig 7). Among them, 26.7 μm is the main peak. The first peak belongs to the category of clay, which is presumed to be formed by chemical deposition or atmospheric dust accumulation in the lake [54]. The second peak corresponds to the EM1 of MGS1, indicating the hydrodynamic deposition process of the lake, but the particle size is finer, indicating that this area is closer to the center of the lake where the hydrodynamic force is weaker. The third peak particle size is 423.8 μm, which belongs to the saltation load. The occurrence of components >200 μm (grain size) in the limnetic phase generally requires sufficient runoff, and its occurrence may indicate the increase of the frequency of heavy rain [54]. The average particle size of EM2 is 43.46 μm, and the mode particle size is 75.5 μm. The EM2 is poorly sorted and negatively skewed. It is mainly composed of silt and sand and occupies a large proportion in the secondary loess phase. This end-member is similar to EM2 in MGS1 to indicate fluid dynamic action, but EM2 in DGS1 has finer particle size, worse sorting and more silt content. In general, the mode particle size of coarse grain components in loess is generally between 12 μm and 80 μm, which represents low-altitude and near-source dust deposition [55]. Therefore, it can be inferred that EM2 materials come from nearby sandy loess, and then are strongly scoured and transported by flowing water, and finally form secondary loess deposition in low-lying areas.

### 5.2 Accumulation process of MGS1 and DGS1 layers

The linear distance between Milanggouwan and Dishaogouwan is only 5317 m, but the sedimentary facies and grain size characteristics of these two sections are significantly different, reflecting the different accumulation processes of the two sites. The results of grain size parameters and end member analysis are discussed in the following.

**Fig 8:**
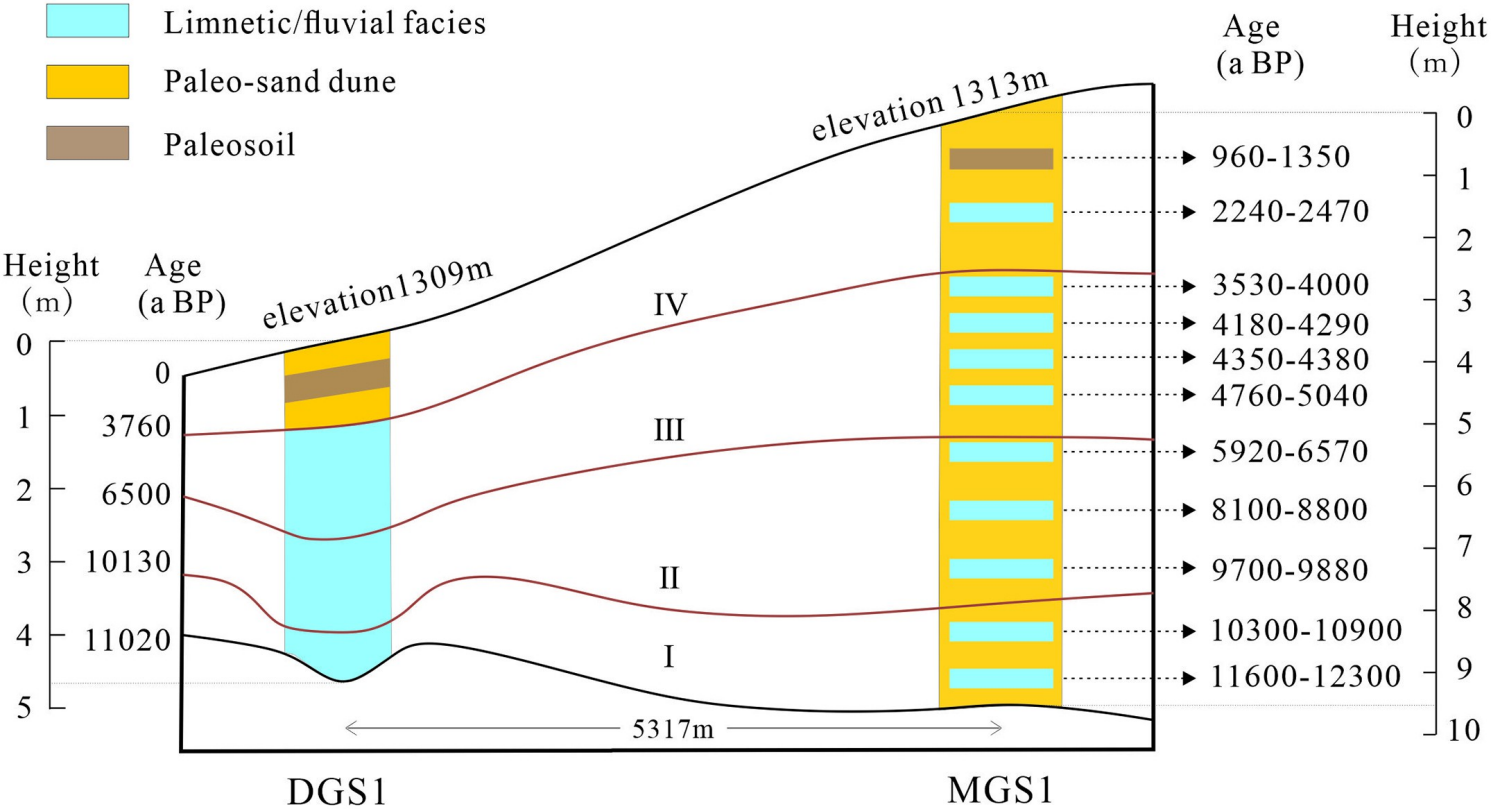
Stage division of MGS1 and DGS1.

In the I stage (11723~10130 aBP) (Fig 8), MGS1 had a coarse average particle size, medium sorting, positive skewness and medium kurtosis. Two fluvial layers, 21FL and 19FL, interbedding with 20D (Figs 1d, 1e and 8). EM2 and EM3 accounted for 46.06% and 47.76%, respectively (Table 4). It can be speculated that MGS1 is located in the microprotrusion terrain, and EM2 is the main flow force and accepts the material deposition of floodplain during the strengthening of summer monsoon, and EM3 is the main wind transport force during the strengthening of winter monsoon. The average particle size of DGS1 was fine but varied greatly, with poor sorting, positive skewness and sharp kurtosis. The silty fine sand, silty very fine sand and silty sand interbedded with small thickness of 30LS~25LS were formed. At this time, the altitude of DGS1 was slightly higher than that of MGS1, and EM2 was the dominant hydrodynamic force. It can be deduced that DGS1 was a small depression in the high place, and EM3 also accounted for a large proportion. Therefore, although the precipitation increased in this period, the intensity was small, and the winter wind sometimes came back. Generally speaking, it can be speculated that the environment began to improve, and the East Asian summer monsoon began to strengthen during this period, which belongs to the early warming period of the Holocene [25].

**Table 4:** Endmember contents of MGS1 and DGS1 at different stages.

Profile	Stage	EM1(%)	EM2(%)	EM3(%)
MGS1	IV	39.15	11.87	48.98
	III	28.67	36.32	35.01
	II	10.87	52.19	36.94
	I	6.18	46.06	47.76
DGS1	IV	16.80	22.87	60.33
	III	45.19	26.37	28.44
	II	43.61	37.37	19.02
	I	22.57	48.92	28.52

In the II stage (10130–6590 aBP) (Fig 8), EM2 and EM3 of MGS1 account for 52.18% and 36.94%, respectively (Table 4), indicating that it is affected by flowing water and aeolian sand accumulation, but flowing water is more intense. Two layers of FL facies (17FL, 15FL), one layer of LS facies (13LS) and three layers of paleo-dune facies (18D, 16D, 14D) were formed. Compared with other stages, the grain size of paleo-dune sand is slightly finer, and the grain size of fluvial facies is coarser, which records better environmental information of Holocene Megatherm. DGS1 was dominated by EM1, accounting for 43.61% (Table 4), and accumulated lacustrine facies with a thickness of about 78cm at 24LS~19LS, in which very fine sand was predominant at 24LS~21LS and silt and clay silt at 20LS~19LS. Mz and σ1 increased, while Sk1 and KG decreased. The grain size showed a trend of thinning, and the sorting degree became worse, indicating that both the area and depth of the lake in DGS1 were increasing, the vegetation coverage around the lake increased, and a large amount of fine particulate matter entered the lake, indicating that the East Asian summer monsoon was strong during this period. Therefore, this period belongs to the peak of the Holocene [29].

In the III stage (6590–3760 aBP) (Fig 8), MGS1 was dominated by EM2 and EM3, forming four layers of paleo-mobile dune sand facies 12D, 10D, 8D, 6D and four layers of LS facies (11LS, 9LS, 7LS, 5LS) interbedding. The grain size parameters fluctuate greatly with the change of sedimentary facies. DGS1 is still dominated by EM1, but EM3 is strengthened, with 12 thin lacustrine layers of 18LS~6LS deposited, mainly interbedded with fine sand, very fine sand and silt, and an ancient mobile dune sand 9FD.Mz and σ1 show a fluctuating trend, Sk1 and KG change sharply, and there are still many peaks and valleys in the same layer. At this time, DGS1 and MGS1 jointly revealed that the environmental changes in this period were relatively fluctuating, and the climate tended to be cold, which was a period of transition to the cold period [32].

In the IV stage (3760~0 aBP) (Fig 8), the average particle size of MGS1 varies greatly, forming two thick layers of paleo-mobile dune sand (4D and 2D), two LS, one D and one MD. The proportion of EM3 is the largest in the whole section. The dynamic conditions of DGS1 changed from EM1 to EM3, and the sediment also changed from the lacustrine to the dune facies. During this period, three layers of paleo semi-fixed/fixed dune sands (5FD, 3FD, 2FD) were included. 1CS, two layers of moving dune sand, 4FD and 0MD. MGS1 is also dominated by EM3, and EM1 accounts for a certain proportion, forming three layers of mobile dune sand, 4D, 2D, 0MD and two layers of LS. The grain size parameters change obviously with different layer phases. The two profiles jointly revealed that the climate changed rapidly and violently during this period, with longer cold-dry time and obvious desertification, which was a period of unstable cooling [32].The secondary sandy loess deposited by DGS1 in 965~1975 aBP correspond with limnetic facies and the dark loessial soil deposited by MGS1 in 960~1350 aBP (Fig 8), indicating that there may be a heavy rain and flood period dominated by EM2 and EM1, which scour the surface dune sand. In the relatively low-lying DGS1 area, the secondary sandy loess was accumulated, while in the area of relatively higher elevation in MGS1, the discontinuous changes of marsh and soil outcropping were formed, and the vegetation cover increased, and the fine-grained material was deposited. Since 960 aBP, the EM3 of MGS1 and DGS1 was absolutely dominant, forming thick modern aeolian sand deposition. It is worth noting that although the D and FD were also dominated for EM3; EM1 and EM2 also accounted for a certain proportion, while the MD deposits was formed by a single dynamic of EM3. It indicates that the source of MD is simpler.

### 5.3 Comparative analysis of Holocene climate fluctuations

**Fig 9:**
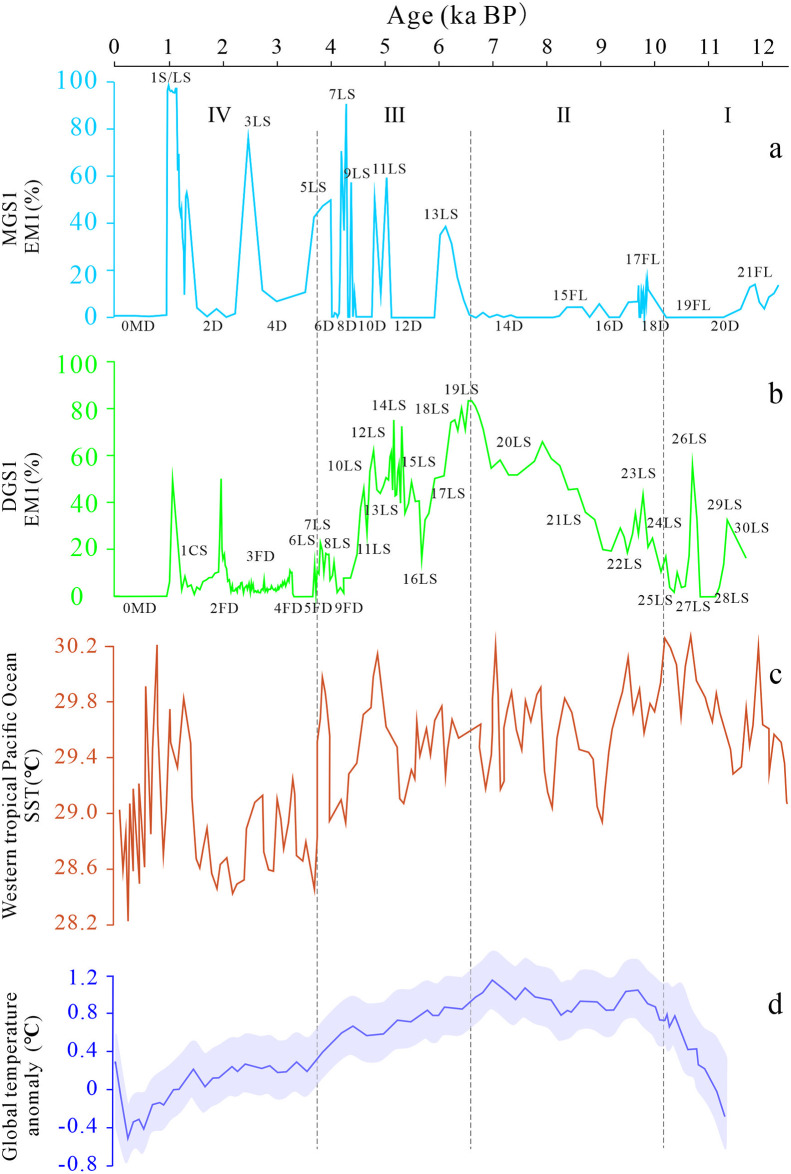
Comparison of EM1 of MGS1(a) and DGS1 (b) with the Western Pacific SST [56] (c) and the reconstructed global temperature (30°N in the Northern Hemisphere) [57] (d) based on climate surrogate indicators.

The EM1 of MGS1 and DGS1 represents a climate environment with sufficient precipitation and increased water inflow into the lake. The EM1 of MGS1 and DGS1 is compared with the paleo-SST (sea surface temperature) in the Western Pacific Ocean [56] and the temperature variation curve of 30°~90°N in the Northern Hemisphere [57] (Fig 9). It is found that the EM1 of MGS1 has a strong fitting relationship with the SST of the Western Pacific Ocean. The 1S/LS, 3LS, 5LS, 11LS, 13LS, 15FL, 17FL, 21FL in MGS1 have a good correspondence with the SST high temperature event of the Western Pacific Ocean, whereas 0MD, 2D, 10D, 12D, 16D and 20D have a good correlation with SST low temperature events in the Western Pacific Ocean (Fig 9a and 9c). The Western Pacific Ocean is the birthplace of the East Asian summer monsoon, and MGS1 has well recorded several large warming and cooling events of the SST in the Western Pacific Ocean, which confirms that the millennium-scale dune sand and fluvial-limnetic interlayers of MGS1 is mainly the result of the fluctuation of cold and warm temperatures dominated by the East Asian winter and summer monsoon [32].

The EM1 of DGS1 fits well with the reconstructed global temperature (30°N in the Northern Hemisphere) trend based on climate surrogate indicators, especially the early Holocene warming period and the Holocene peak period, from the beginning of the Holocene warming period to the peak of the Holocene warm temperature, and the two trends are almost identical (Fig 9b and 9d). There is also a good correspondence between the fluctuation period of the transition from the warm to the cold period and the unstable period of the cooling and the frequent change of the desertification, indicating a trend of temperature decline. In general, because DGS1 is located at a lower altitude than MGS1, its fluctuation record is more obvious, such as 26LS, 19LS and 3FD. In addition, the humid period represented by 1CS in the secondary loess layer is weakly recorded in the Northern hemisphere temperature curve, which may be related to the fact that Salawusu is located in the boundary zone (Fig 1a) and is more sensitive to climate change.

## 6 Conclusions

In this paper, the MGS1 section of Milanggouwan and DGS1 section of Dishaogouwan were selected as the comparative research objects. The grain size content, grain size parameters and end-member model were analyzed for the two sections. The Holocene climate fluctuation relationship recorded between different sedimentary facies in the Salawusu River Valley was discussed, and certain conclusions were drawn as follows:

1. The mixed range of sand, silt and clay in DGS1 also has a certain distribution, indicating that the same section of DGS1 may be mixed with substances of various dynamic processes. The particle size parameters showed that the mean particle size, standard deviation, skewness and kurtosis of MGS1 were smaller than those of DGS1, which indicates that MGS1 has coarser particles, better sorting performance, more symmetrical skewness and flatter peak state. The paleodune sand facies in the upper part of DGS1 profile has coarser particle size, better sorting and sharp peak state. The lacustrine phase in the lower part of the section has smaller particle size, poor sorting and relatively flat peak state. MGS1 sediments are mainly distributed in sand and silty sand in DGS1.
2. End-member analysis was used to separate endmember components from MGS1 and DGS1 grain size data, and the three endmember components selected represent different sedimentary dynamics and transport characteristics. EM1 in MGS1 was mainly derived from terrigenous detritus, and then entered the lake through the mixing of flowing water and wind and became lakebed deposition. EM2 indicates floodplain deposits with weak hydrodynamic conditions. EM1 in DGS1 indicates different lake hydrodynamic deposition, EM2 indicates the deposition formed by strong flushing and transport of flowing water; and EM3 of both profiles together indicates the accumulation of aeolian sand formed during wind transport.
3. The accumulation process of MGS1 and DGS1 can be divided into four periods: early warming period (I, 11700~10130 aBP), Holocene peak period (II, 10130~6590 aBP), transition period to cold period (III, 6590–3760 aBP) and unstable cooling period (IV, 3760~0 aBP). The EM1 of MGS1 has a strong fitting relationship with the SST of the Western Pacific Ocean, especially has a good corresponding relationship with the SST high temperature events of the Western Pacific Ocean. The EM1 of DGS1 has a high fitting degree with the trend of global temperature reconstructed based on climate surrogate indicators.

## Supporting information

S1 Fig Comparison of facies and particle size parameters between MGS1 and DGS1 layers. Detailed information of a~h please refer to the text.
(XLS)

S1 Data (XLS)

## References

[1] Chen FH, Fu B, Xia J, Wu D, Wu S, Zhang Y, et al. Major advances in studies of the physical geography and living environment of China during the past 70 years and future prospects. Sci. China Earth Sci. 2019;62: 1665–1701.

[2] Qin DH. Introduction to Climate Change Science. Beijing: Science Press; 2018.

[3] Zhang DE. High resolution paleoclimatic records available from Chinese historical documents. Quaternary Sci. 1995;15: 75–81.

[4] Pang JL, Huang CC, Liu AN, Wang LJ. Distribution characteristics and significance of some elements in Holocene loessal-paleosol sequence in southern Loess Plateau. Quaternary Sci. 2007;27: 357–364.

[5] Ding M, Pang JL, Huang CC, Li YH, Huang LJ, Niu XL. Geochemical characteristics of major sequence elements in Holocene loess paleosoil of eastern Guanzhong. J. of Desert Research. 2011;31: 862–867.

[6] Lombardo U, Rodrigues L, Veit H. Alluvial plain dynamics and human occupation in SW Amazonia during the Holocene: a paleosol-based reconstruction. Quaternary Sci. Rev. 2018;180: 30–41.

[7] Chen FH, Zhu Y, Li JJ, Shi Q, Xi LY. Wunemann. B. Rapid changes of Holocene millennial-scale summer monsoon recorded by lacustrine sediments in Minqin Basin. Chinese Sci. Bull. 2001;46: 1414–1419.

[8] Jiang QF, Shen J, Liu XQ, Zhang EL, Xiao XY. High-resolution paleoclimatic evolution of lacustrine sedimentary records since Holocene in the Westerly region. Chinese Sci. Bull. 2007;52: 1042–1049.

[9] Hopley CA, Jones BG. Holocene evolution and depositional model of a bayhead delta, lake Illawarra, Australia. Sediment. J. of the Int. Association of Sediment.2022;69: 1927–1952.

[10] Bianchi GG, Mccave IN. Holocene periodicity in North Atlantic climate and deep-ocean flow south of Iceland. Nature. 1999;397: 515–517.

[11] Duan KQ, Yao TD, Wang NL, Xu BQ, Thompson LG. The unstable Holocene climatic change recorded in an ice core from the central Tibetan Plateau. Sci Sin Terrae. 2012;42: 1441–1449.

[12] Sigl M, Toohey M, McConnell JR, Cole-Dai J, Severi M. Volcanic stratospheric sulfur injections and aerosol optical depth during the Holocene (past 11 500 years) from a bipolar ice-core array, Earth Syst. Sci. Data. 2022;14: 3167–3196.

[13] Zhou WJ, Lu XF, Wu ZK, Wu KZ, Deng L, Jull AJ, et al. Peat records and accelerator radiocarbon dating of Holocene climate change in the Zoige Plateau. Chinese Sci. Bull. 2001;46: 1040–1044.

[14] Rao ZG, Shi FX, Li YX, Huang C, Zhang XZ, Yang W, et al. Long-term winter/summer warming trends during the Holocene revealed by α-cellulose δ18O/δ13C records from an alpine peat core from central Asia. Quaternary Sci. Rev.2020;232: 106217–106228.

[15] Bysouth D, Finkelstein SA. Linking testate amoeba assemblages to paleohydrology and ecosystem function in Holocene peat records from the Hudson Bay lowlands, Ontario, Canada. The Holocene. 2021;31: 457–468.

[16] He YQ, Wang YJ, Kong XG, Chen H. δ18O records from a high-resolution cave stalagmite in Dongge Cave, Guizhou Province, China during the past 1000 years. Chinese Sci. Bull. 2005;50: 1114–1118.

[17] Shao XH, Wang YJ, Cheng H, Shun XG., Wu JY. Holocene monsoon climate evolution and drought events from Stalagmite records in Shennongjia, Hubei Province. Chinese Sci. Bull. 2006;51: 80–86.

[18] Burstyn Y, Shaar R, Keinan J, Ebert Y, Ayalon A, Bar-Matthews M. Holocene wet episodes recorded by magnetic minerals in stalagmites from Soreq cave, Israel. Geology. 2022;50: 284–288.

[19] Shao XM, Huang L, Liu HB, Liang EY, Fang XY, Wang LL. Tree-ring records of precipitation in Delingha region, Qinghai Province. Sci. China Ser. D.2004;34: 145–153.

[20] Li Q, Liu Y, Deng RL, Liu RS, Song HM, Wang Y, et al. Combination of Tree Rings and Other Paleoclimate Proxies to Explore the East Asian Summer Monsoon and Solar Irradiance Signals: A Case Study on the North China Plain. Atmosphere. 2020;11: 1180–1194.

[21] Pearl JK, Anchukaitis KJ, Donnelly JP, Pearson C, Zimmermann GL. A late Holocene subfossil Atlantic white cedar tree-ring chronology from the northeastern United States. Quaternary Sci. Rev. 2020;228: 106104.

[22] Niu DF, Li BS, Du SH, Wen XH, Qiu SF, Xian J, et al. Cold events of Holocene indicated by primary elements distribution of the high-resolution sand dunes in the Salawusu River Valley. J. Geogr. Sci. 2008;18: 26–36.

[23] Liu K, Lai ZP. Chronology of Holocene sediments from the archaeological Salawusu site in the Mu Us Desert in China and its paleoenvironmental implications. J. of Asian Earth Sci. 2012;45: 247–255.

[24] Si YJ, Li BS, Zhang DD, Wang FN, Wen XH, Guo YJ. Climate fluctuation record from China’s Salawusu RIVER valley during the early last glacial. Geochem Int+. 2013;51: 240–248.

[25] Niu DF, Li BS, Wen XH, Du SH, Li ZW, Wang FN Si, et al. The Holocene Ka-Scale climate variation indicated by trance elements of the MGS1 segment in the Salawusu River Valley, China. Acta Geol Sin-Engl. 2011;85: 300–308.

[26] Niu DF, Li BS, Wang FN, Chen Q, Shu PX, Wen XH, et al. Holocene climate fluctuations from the record of elements in the Mu Us Desert: evidence from the DGS1 segment of the Salawusu River Valley. Acta Sediment. Sin. 2015;33: 735–743.

[27] Niu DF, Li BS, Wei JG, Wen XH, Shu PX, Si YJ. Holocene millennial-scale climate variations as recorded by Rb and Sr concentrations for the MGS1 stratigraphical segment of Milanggouwan section in the Salawusu River Valley of Southeast Mu Us Desert. Geochemistry-Germany. 2016;45: 155–163.

[28] Wang FN, Li BS, Niu DF, Li ZW, Wen XH, Si YJ, et al. Holocene millennial scale variations from records of Grain size and CaCO3 in MGS1 segment of Mianggouwan Section in the Salawusu River Valley, China. J. of Desert Research. 2012;32: 331–339.

[29] Wang FN, Li BS, Niu DF, Wen XH, Li ZW, Si YJ, et al. Holocene climate change recorded by CaCO3 in the DGS1 segment in the southeast of the Mu Us Desert, China. J. of Earth Env. 2015;6: 145–153.

[30] Zhao Q, Fan R, Li BS, Mischke S, Zhang CJ. Geochemical responses to paleoclimate: evidence from the early-mid Holocene lake deposits at Dishaogouwan section in the Salawusu catchment of inner Mongolia. Marine Geol & Quaternary Geol. 2013;33: 103–112.

[31] Shao YJ. Pollen component and paleoclimate and paleovegetation in Sjara-Osso-Gol River since late Pleistocene Epoch. J. of Dessert Research. 1987.07: 22–28.

[32] Shu PX, Li BS, Niu DF, Wang FN, Wen XH, Si YJ, et al. Climate variations recorded by the grain-size from the DGS1 segment in the southeast of China’s Mu Us Desert during the Holocene. Sci. Geographica Sin. 2016;36: 448–457.

[33] Qi GQ. Quaternary mammalian fossils from Salawusu River district, Nei Mongol. Vertebrata Palasiatica. 1974;13: 239–249.

[34] Xie JY, Gao SY, Dong GR, Li BS. Zoomcoenosium in Sara Wusu. Journal of Desert Research. 1995;15: 313–322.

[35] Weltje GJ. End-member modeling of compositional data: Numerical-statistical algorithms for solving the explicit mixing problem. Mathematical Geol. 1997;29: 503–549.

[36] Paterson GA, Heslop D. New methods for unmixing sediment grain size data. Geochemistry. 2015;16: 4494–4506.

[37] Liu MH, Li XS, Han ZY, Wang YC, Yuan XK, Ren YC. Parametric end-member analysis of the grain size distribution of the Xiashu loess and its provenance tracing. J. of Earth Env. 2021;12: 510–525.

[38] Zhang W, Chang R, Ma RF, Ma HN, Ge RZ, Shun B, et al. Grain-size and chemical characteristics of the lacustrine deposits from the Xi Tuanpiao profile in Jinzhou, southern Liaoning province and their environmental significances. Marine Geol. & Quaternary Geol. 2020;40: 193–205.

[39] Michel MM, Samara CG, Maria CS, Rodrigo AU, Bianca SM, Paulo AL, et al. Grain-size end-members and environmentally sensitive grain-size components: A comparative study in the mud shelf depocenters off southern Brazil. Int. J. Sediment. Res. 2021;36: 317–327.

[40] Zhan JZ. Sedimentary characteristics, physicochemical properties and environmental evolution of Houtian section in northern Jiangxi during the last glacial period. M.Sc. Thesis, the East China University of Technology. 2021. https://cdmd.cnki.com.cn/Article/CDMD-10405-1021852164.htm

[41] Zhu BH. Climate of China. Science Press; 1962.

[42] Dong GR, Li BS, Chen YZ. Comprehensive Study on Late Quaternary Geology and Hominins in Salawusu River. Science Press; 2017.

[43] Folk PL, Ward WD. Brazos Reviver bar: A study in the significance of grain size parameters. J. of Sediment Petrol. 1957;27: 3–26.

[44] Hateren JA, Prins MA, Balen RT. On the genetically meaningful decomposition of grain-size distributions: A comparison of different end-member modelling algorithms. Sediment. Geol. 2018;375: 49–71.

[45] Ding XG, Ye SY, Gao ZJ. Development and applications of grain size analysis technique. Global Geol. 2005;24: 203–207.

[46] Xu LY, Yang LH, Zhang S, Zhai TC. End-member analysis of grain size of reticulated red clay and its indication significant section of Xuancheng. Earth and Environment. 2021;49: 646–654.

[47] Zhao GG, Ting QC, Du WX, Pei Y, Er QY. End member model analysis of grain size for the loess in Linfen Basin, China. Marine Geol & Quaternary Geol. 2021;41: 192–200.

[48] Shepard FP. Nomenclature based on sand-silt-clay ratios. J. of Sediment. Geol. 1954;24: 151–158.

[49] Yin ZQ, Qin XG, Wu JS, Ning B. The multimodal grain-size distribution characteristics of Loess, desert sand, lake and river in Northern China. Acta Sediment Sin. 2009;27: 343–351.

[50] Bai M, Lu RJ, Din ZY, Wang LD. Analysis of endmembers of Grain size in the Eastern Sandy Land of Qinghai Lake and its indicative significance. Quaternary Sci. 2020;40: 1203–1215.

[51] Li ZP, Yue LP, Xue XX, Wang M, Yang LR, Nie HG, et al. Characteristics of Sandy Desertification grain size and its geological significance in the Southeastern margin of Mu Us Sandy Land. Acta Sediment. Sin. 2006;24: 267–275.

[52] Liu DY, Li WR, Peng SS, Wang L. Current application of grain analysis in Chinese Loess paleoclimatic study. J. Ocean U. China (Natural Science Edition). 2010;40: 79–84.

[53] Song J, Chun X. The Spatial Variation and Grain size character of different land cover types in the Ulanbuh Desert. J. of Desert Research. 2018;38: 243–251.

[54] Sun DH, An ZS, Su RX, Wu XM, Wang SM. A mathematical method for the separation of particle size components from sediments in Paleoenvironment and its application. Prog. Nat. Sci. 2001.;20: 47–54.

[55] Liu T, Yang XP, Dong JF, Fan XY, Li HW, Zhu BQ. A preliminary study of relation between Megadune Shape and Wing Regime in the Badain Jaran Desert. J. of Desert Research. 2010;30: 1285–1291.

[56] Stott L, Cannariato K, Thunell R, Haug GH, Koutavas A, Lund S. Decline of surface temperature and salinity in the western tropical Pacific Ocean in the Holocene epoch. Nature. 2004;431: 56–59. doi: 10.1038/nature02903 15343330

[57] Shaun AM. Jeremy DS. Peter UC. Alan CM. A Reconstruction of Regional and Global Temperature for the Past 11300 Years. Science. 2013;339: 1198–1201. doi: 10.1126/science.1228026 23471405

